# Editorial: Impacts of VEMP and VHIT on the diagnosis of vestibular diseases

**DOI:** 10.3389/fneur.2023.1244367

**Published:** 2023-07-10

**Authors:** Alexander Andrea Tarnutzer, Toru Seo, Chisato Fujimoto, Dominik Straumann, Toshihisa Murofushi

**Affiliations:** ^1^Department of Neurology, Cantonal Hospital of Baden, Baden, Switzerland; ^2^Faculty of Medicine, University of Zurich, Zurich, Switzerland; ^3^Department of Otolaryngology, St. Marianna University Yokohama Seibu Hospital, Yokohama, Japan; ^4^Department of Otolaryngology and Head and Neck Surgery, Graduate School of Medicine, The University of Tokyo, Tokyo, Japan; ^5^Department of Neurology, University Hospital Zurich, Zurich, Switzerland; ^6^Department of Otolaryngology, Teikyo University School of Medicine, Mizonokuchi Hospital, Kawasaki, Japan

**Keywords:** vestibular, vestibular-evoked myogenic-potentials (VEMPs), video head impulse test, acute vestibular syndrome (AVS), acute peripheral vestibulopathy, state-of-the-art research

With the introduction of quantitative testing of both the angular (i.e., the semicircular canals) and linear (utriculus and sacculus) acceleration sensors in the inner ear and their neural afferents, vestibular mapping has become broadly available [e.g. (1)]. Important milestones were laid by Colebatch et al. (2), who introduced cervical vestibular-evoked myogenic-potentials (cVEMPs) for assessing saccular function, and about a decade later, when the basics of ocular VEMPs (oVEMPs) for assessing utricular function were described by Rosengren et al. (3). Likewise, with the development of a lightweight high-resolution video head impulse testing (vHIT) device, the Sydney team led by Curthoys, Halmagyi, MacDougall, McGarvie, and Weber introduced quantitative video-based testing of the horizontal angular vestibulo-ocular reflex (aVOR) in 2009 (4). Four years later, testing of the vertical semicircular canals using vHIT was proposed by the same group (5, 6).

Several vHIT systems are now commercially available. Quantitative testing of the aVOR became a cornerstone in the diagnostic workup of patients with dizziness/vertigo or balance disorders, both in the emergency department and on the ward, but also in specialized private practices and in tertiary vertigo clinics. The impact of quantitative assessments of vestibular function is significant in patients with acute vestibular syndrome, as demonstrated by Korda et al. (7) and Nham et al. (8), who reported increased diagnostic accuracy for detecting vertebrobasilar stroke by quantitative testing compared with bedside testing. Likewise, vHIT has significantly improved the diagnosis of acute peripheral vestibulopathy, especially in cases with an isolated involvement of the inferior branch of the vestibular nerve (9) or the posterior semicircular canal (10, 11). Additionally, in telemedicine approaches with acutely dizzy patients, vHIT plays an important role, as demonstrated in a recent feasibility study (12).

Thus, this Research Topic aimed to bring together new insights into the clinical utility of quantitative vestibular testing, focusing on current state-of-the-art technologies, such as vHIT and VEMPs. A highlight of this Research Topic is a series of manuscripts that discuss various factors that may influence the aVOR as recorded by vHIT. In an in-depth review, the use and interpretation of vHIT results in the clinical setting are critically discussed, focusing on the geometrical basis and the underlying principles when collecting aVOR responses (Curthoys et al.). This review highlights factors that can affect the eye-velocity responses recorded by the goggles, including the orientation of the goggles on the head, the amount of head pitch, and the contribution of the vertical canals to the horizontal canal response. Similarly, in a systematic review on factors affecting the variability in the aVOR gain of vHIT in healthy human subjects, participant-based factors, tester/examiner-based factors, protocol-based factors, and equipment-based factors were identified as the main categories that lead to significant variation (Money-Nolan and Flagge). Based on their findings, the authors proposed strategies for reducing aVOR gain variability in clinical practice, including the retrieval of individual normative values, sufficient training in applying and interpreting vHIT responses, and analyzing the entire traces and not just focusing on the reported aVOR gain values. In another study, the clinical characteristics of corrective saccades in patients with normal vHIT gain were investigated (Kabaya et al.). Based on their observation that the side with catch-up saccades (peak-velocity >100°/s) had a significantly smaller aVOR gain and a smaller caloric response, the authors proposed that catch-up saccades with a normal aVOR gain could be a sensitive indicator of mild semicircular canal dysfunction.

Several manuscripts in this Research Topic focused on the integrity of the vestibular system in patients with specific disorders, such as SARS-CoV-2, persistent postural-perceptual dizziness (PPPD), and Menière's disease (MD), or in especially vulnerable populations, such as children. With dizziness being a frequent complaint in patients suffering from SARS-CoV-2, vHIT and VEMP data in 50 patients diagnosed with SARS-CoV-2 were analyzed (Zaubitzer et al.). After finding no evidence of a significantly reduced semicircular canal or otolith function, the authors concluded that a persistent structural affection of the vestibular system by SARS-CoV-2 seems to be unlikely. When comparing vestibular responses in patients with definite unilateral MD with migraine headaches or without headaches, a male predominance in the group without headaches and a higher rate of severe saccular dysfunction for cVEMPs in patients with MD and migraine headaches than in MD patients without headaches were reported (Inui et al.). Thus, the presence of migraine headaches may affect the pattern of vestibular function in MD. How coexisting deficits due to preceding vestibular disorders affect PPPD symptoms is not well understood. Characterizing the clinical features of PPPD with or without vestibular dysfunction using both VEMPs and vHIT and various questionnaires, including the DHI, was therefore pursued in another study (Azami et al.). Interestingly, significantly higher (i.e., worse) DHI scores were noted in those PPPD patients with combined utricular and saccular damage, emphasizing the importance of quantifying otolith function in PPPD when deciding on treatment strategies.

A systematic review and meta-analysis was performed to advance our understanding of the etiology and prevalence of vertigo disorders in children to eventually improve the diagnostic approach and management of pediatric patients presenting with dizziness or vertigo (Zhang et al.). With peripheral disorders identified in 52% of all patients and central disorders in 29%, the most frequently made diagnoses were vestibular migraine and BPPV of childhood (20% each). Reported gender specificity in vestibular migraine and psychogenic vertigo emphasizes the importance of an individualized approach. Recovery or improvement after symptomatic treatment and non-pharmacological treatment point to the most suitable management strategies in this review.

In summary, this Research Topic further emphasizes the clinical utility of state-of-the-art vestibular testing in vestibular disorders and sheds new light on the pearls and pitfalls in vHIT recording and interpretation and VEMP findings in various disorders. At the same time, it highlights current needs and future research directions in the field, such as gaining more knowledge about diagnosing and treating vertigo and dizziness in childhood and the interplay between vestibular migraine and MD.

## Author contributions

AT drafted the editorial. TS, CF, DS, and TM critically reviewed and edited the editorial. All authors approved the submitted manuscript.
